# Knowledge of Stroke Risk Factors and Warning Signs in Patients with Recurrent Stroke or Recurrent Transient Ischaemic Attack in Thailand

**DOI:** 10.1155/2017/8215726

**Published:** 2017-10-09

**Authors:** Jittima Saengsuwan, Pathitta Suangpho, Somsak Tiamkao

**Affiliations:** ^1^Department of Rehabilitation Medicine, Faculty of Medicine, Khon Kaen University, Khon Kaen, Thailand; ^2^Faculty of Nursing, Vongchavalitkul University, Nakhon Ratchasima, Thailand; ^3^Department of Medicine, Faculty of Medicine, Khon Kaen University, Khon Kaen, Thailand; ^4^Northeastern Stroke Research Group, Khon Kaen University, Khon Kaen, Thailand

## Abstract

Stroke is a global burden. It is not known whether patients who are most at risk of stroke (recurrent stroke or recurrent transient ischaemic attack) have enough knowledge of stroke risk factors and warning signs. The aim of this study was to assess the knowledge of stroke risk factors and warning signs in this high-risk population. We performed a cross-sectional questionnaire-based study of patients with recurrent stroke or recurrent TIA admitted to Srinagarind Hospital and Khon Kaen Hospital, Thailand. A total of 140 patients were included in the study (age 65.6 ± 11.3 years [mean ± SD], 62 females). Using an open-ended questionnaire, nearly one-third of patients (31.4%) could not name any risk factors for stroke. The most commonly recognized risk factors were hypertension (35%), dyslipidemia (28.6%), and diabetes (22.9%). Regarding stroke warning signs, the most commonly recognized warning signs were sudden unilateral weakness (61.4%), sudden trouble with speaking (25.7%), and sudden trouble with walking, loss of balance, or dizziness (21.4%). Nineteen patients (13.6%) could not identify any warning signs. The results showed that knowledge of stroke obtained from open-ended questionnaires is still unsatisfactory. The healthcare provider should provide structured interventions to increase knowledge and awareness of stroke in these patients.

## 1. Introduction

Stroke is one of the leading causes of adult disability worldwide [1, 2]. In Thailand, stroke is the third leading cause of death [3] with approximately 250,000 patients suffering from stroke each year [4]. Stroke causes disability, but this may not be the only effect of stroke: stroke recurrence, which has the cumulative risk of 25% in 5 years, increases the risk of severe disability and death [5].

Recent evidence has shown that the administration of recombinant tissue plasminogen activator (rtPA) in patients with recurrent stroke improves neurological outcomes [5]. A candidate for rtPA must arrive at the hospital without delay. Delayed arrival may be due to a lack of patient and public awareness of stroke symptoms, a decision to take a wait-and-see attitude, and a lack of proper immediate action [6, 7]. More knowledge of stroke risk factors and stroke warning signs amongst patients is associated with an increased probability of correctly calling emergency services [8].

After stroke, it would be expected that patients would have more knowledge of stroke risk factors and symptoms. However, studies showed that this is not the case [9]. Most stroke patients and their carers had inadequate understanding of stroke, such as the causes of stroke or preventive measures [10, 11]. Studies demonstrated that previous stroke had no impact on knowledge of stroke. Moreover, knowledge of stroke in patients after stroke or TIA was as low as in randomly selected healthy individuals [12]. As a consequence of limited knowledge, stroke patients may not change their unhealthy prestroke lifestyles to reduce their risk of recurrent stroke [13] and may not be able to recognize stroke warning signs if it happens a second time. This leads to the same problem of delayed hospital arrival and loss of another chance of having proper standard treatment.

Most studies on the knowledge of stroke risk factors and warning signs were done in patients after first-ever stroke or transient ischaemic attack (TIA), but there are limited data on patients with recurrent episodes of stroke or TIA. Thus, the aim of this study was to assess the knowledge of stroke risk factors and stroke warning signs in this high-risk population.

## 2. Methods

### 2.1. Study Design and Participants

This cross-sectional study was approved by the Ethics Review Committee of Khon Kaen University, Thailand (ref. HE571111). All subjects gave written informed consent before participating in the study.

Patients with recurrent stroke or recurrent TIA were recruited from both in-patient and out-patient units in Srinagarind Hospital and Khon Kaen Hospital, which are tertiary hospitals in the northeast of Thailand, from April to July 2014. Patient inclusion criteria were (1) a diagnosis of recurrent stroke or recurrent TIA confirmed by radiological evidence, (2) age more than 18 years, (3) sufficient cognitive and communicative ability, and (4) a willingness to participate in the study. The exclusion criteria were (1) severe aphasia limiting comprehension, (2) medical history of dementia, and (3) inability to give informed consent.

### 2.2. Measures

The details of demographic and clinical variables such as stroke type and side were obtained from medical records. Data on stroke knowledge were collected by face-to-face interview by trained registered nurses. The questionnaire used in this study was developed in 2011 and comprises stroke knowledge, risk factors, stroke warning signs, and the treatment of stroke [14]. This questionnaire was validated in first-ever stroke patients. The first part consists of open-ended questions and the second part contains closed-ended questions. The analyses in this article focused on the questions on (1) general stroke knowledge (definition of TIA and stroke), (2) stroke risk factors, and (3) stroke warning signs. In the closed-ended questionnaire, the maximum total score was 17 points. For every correct answer, 1 point was added.

### 2.3. Statistical Analysis

Data on the response to each questionnaire item and other categorical variables are presented as frequencies and percentages. Mean and standard deviations are presented for continuous variables. Normality of data was examined using Kolmogorov-Smirnov tests. Since the data were found not to be normally distributed, Mann–Whitney *U* tests were used to analyze group differences within the sum of the scores from closed-ended questions on patient knowledge of stroke definition, stroke risk factors, and stroke warning signs and other variables (age group, gender, educational level, and disability level). *p* < 0.05 was considered to be statistically significant. The analyses were performed using SPSS (Version 20.0, IBM Corp., Armonk, NY).

## 3. Results

Of the 140 patients included (age 65.6 ± 11.3 years [mean ± SD], 62 females), most had a low educational level. The most common recurrent episode was ischaemic stroke, which was diagnosed in 119 patients (85.0%) (Table 1).

Most patients had information about stroke after their illness (86.4%). The main sources of information were from healthcare professionals (from nurses, 76.4%, and from doctors, 69.3%). Most patients rated their lack of knowledge of stroke from moderate to high (Table 2). 20 patients (14.3%) felt that they had no lack of knowledge. Most patients wanted to know more about rehabilitation, diet, cause and clinical course of stroke, and exercise.

### 3.1. Stroke Knowledge Results from the Open-Ended Questionnaire

Approximately one-quarter of patients had no idea which organ was involved in stroke, and a similar proportion of patients correctly identified that stroke is a problem in the brain. Nearly half (44.3%) could not define TIA and approximately one-third (32.1%) could not define stroke. Less than 4% of patients could correctly define TIA as a temporary disruption of blood supply to the brain. Patients most commonly defined stroke as inadequate brain perfusion (34.2%) (Table 3).

A quarter of patients (25%) could not name any risk factors for stroke. The most commonly identified risk factors were hypertension (35%), dyslipidemia (28.6%), and diabetes (22.9%). The average number of risk factors they could recall was 1.4. Only 17.1% of patients named three or more risk factors. Regarding stroke symptoms, the most commonly recognized warning signs were sudden unilateral weakness (61.4%), sudden trouble with speaking (25.7%), and sudden trouble with walking, loss of balance, or dizziness (21.4%). Nineteen patients (13.6%) could not identify any warning signs.

Slightly more than half of patients who had underlying hypertension recognized hypertension as one of the risk factors (53.6%). Only 25% of patients with diabetes and 11.7% of patients with dyslipidemia identified diabetes and dyslipidemia as risk factors.

### 3.2. Stroke Knowledge Results from Closed-Ended Questionnaires

Knowledge of stroke increased dramatically when a closed-ended questionnaire was used (answer: true or false). Most patients correctly defined TIA and stroke. The most commonly identified risk factors were hypertension (96.4%), dyslipidemia (96.4%), and aging (94.3%). The most commonly identified warning signs were sudden unilateral weakness (98.6%), sudden unilateral numbness (97.9%), and trouble with walking, loss of balance, or dizziness (92.1%) (Table 4).

### 3.3. The Relation between the Sum of Knowledge Scores from Closed-Ended Questions

Patients with a higher degree of disability, characterised by a Barthel index lower than 60, had a significantly lower score compared with those with a higher Barthel index (mean difference of 1.1). There was no statistically significant difference in knowledge in terms of age group, gender, living situation, and education level (Table 5).

## 4. Discussion

This study aimed to assess knowledge of stroke risk factors and warning signs in patients with recurrent stroke. This study revealed that knowledge of stroke in patients with recurrent stroke was insufficient. Only one-quarter (25%) of patients correctly identified stroke as a disorder of the brain, which is similar to that reported in a normal population (24%) and is at the lower end of the range previously reported in stroke (26–52.4%) [10, 15–17].

Based on the open-ended questionnaire, a quarter of patients (25%) could not name any risk factors for stroke. The most commonly identified risk factors were hypertension, dyslipidemia, and diabetes. These findings are comparable to previous studies using open-ended questionnaires in first-stroke patients: 8–52% of stroke patients could not name any stroke risk factors [17–20]. The commonly recalled risk factors in stroke patients were hypertension (19.5–48%), smoking (11–48%), and dyslipidemia (10–27%). The poorly recalled risk factors were diabetes (4–8%) and heart disease (3–7.5%) [17, 18, 20–22]. Our results showed that no one could recall heart disease as stroke risk factor; however, nearly a quarter of patients could identify diabetes as a risk factor (22.9%). The average number of risk factors named was 1.4, which is lower than in previous studies in stroke patients (2.5) [17].

Based on Thai national data, the most common risk factors identified in stroke patients are hypertension (57%), smoking (27%), and diabetes (24%) [23]. Interestingly, nearly half of patients with hypertension and a majority of patients with diabetes or dyslipidemia could not name these diseases as risk factors for stroke. The failure to identify their own risk factors may lead to a continuation of unhealthy lifestyle or poor medical compliance leading to a higher chance of recurrent stroke. On the other hand, patients who recognized their future risk of stroke were more likely to pursue a healthy lifestyle [20]. Based on the scientific knowledge that implementation of secondary preventive measures in stroke patients—namely, dietary modification, exercise, aspirin, statin, and an antihypertensive agent—can theoretically reduce the relative risk of recurrent vascular events by 80%, the risk of recurrent stroke should be greatly reduced in patients who increase their medical compliance and modify their behaviour [24]. All patients failed to identify previous TIA or stroke as a risk factor [22].

Slightly more than one-tenth of patients (13.6%) could not identify stroke warning signs. This finding was similar to the finding of Weltermann et al. in stroke support-group members (9.8%) but lower than in the study of Slark et al. (34%) who did a survey on 622 stroke survivors in the UK [17, 20]. The most commonly identified stroke warning signs were sudden numbness or weakness of the face, arm, or leg and the least identified warning signs were a sudden visual problem; this is consistent with a previous study [21].

The discrepancies in the responses of patients from open-ended and closed-ended questionnaires were discussed previously [6], and this study confirmed these findings. Results from closed-ended questionnaires showed that there was a dramatic increase in the ability to recognize the definitions of stroke and TIA, stroke risk factors, and stroke warning signs. The results obtained from the open-ended questionnaire may be more reliable as they better reflect real-life situations.

The results regarding the influence of age group, gender, educational level, living situation, and disability level on stroke knowledge scores showed that patients with higher stroke disability had significantly lower stroke knowledge. This has not been documented previously and we propose that it may be related to lower cognition in more severely disabled patients; this hypothesis requires further investigation. Previous studies demonstrated that younger stroke patients and patients with higher education level had higher stroke knowledge [22, 25]. That this was not seen in the present study may be due to the lower sample size.

It is important to note that although more than three-quarters of recurrent stroke patients reported having information about stroke from healthcare professionals, the knowledge about stroke was still unsatisfactory. These figures suggest that information or the method to provide information from healthcare professionals is probably not appropriate to the educational levels of patients. Health professionals may not take into account communication barriers with the patients to ensure that patients understand and retain all health-related educational messages that are given. This fact is important as if patients were aware of the risk factors, they were more likely to modify their lifestyle [13]. Health professionals should consider risk factor education as a tool to improve medical adherence and lifestyle modification for secondary prevention strategies.

Improving the provision of information after stroke was shown to improve outcomes. There are several possible reasons for this. Firstly, improvement in stroke knowledge increases satisfaction and decreases depression and anxiety symptoms [26]. Secondly, if patients have an accurate perception of their risk factors, they are more likely to modify their behaviour to prevent the disease [13]. And thirdly, increasing stroke knowledge may result in earlier arrival in the emergency department because of early stroke recognition. In order to increase self-management of this high-risk group patients, the communication of risk factors and behavioural modification should be strongly emphasized. The low level of knowledge found in this study clearly indicates that there is an urgent need to provide a national policy for providing better educational system for patients after stroke.

As stroke is a chronic disease and the information required varies over time, the way to educate stroke patients and their carers should be well structured. The possible reasons for an unsuccessful educational system may be as follows: (1) patient factors (e.g., at the time of hospitalization the patients might be stressed and unable to pay full attention to the information given at that time and some patients may in addition have concentration or memory problems [27]); (2) care provider factors (e.g., the information may not be given to stroke patients in some cases and there may not be a person responsible for giving the information [28]); (3) information factors (since most patients reported an inadequate quantity of information [28]). The information should also be chronologically given to suit the different phases of stroke and adjusted to underlying diseases, level of disability, and requirements. Information booklets and leaflets may supplement education [27]; and (4) methods of education; for example, repeated information, having more interaction, or giving patients opportunities to solve the problem and give feedback may be more effective in improving knowledge [29]. Importantly, the education method may also need to be individualized because of age and cognitive ability. Educational background influences a patient's preferred mode of information gathering [30]. Additionally, important aspects that influence the behaviour of stroke patients such as social, cultural, and economic context that the patients are in should be taken in mind of the healthcare provider as this accounted for the patient's lifestyle and awareness of disease.

The limitations of our study include having specific inclusion and exclusion criteria, thus limiting the generalizability of the results in the overall recurrent stroke population. For example, we have excluded patients with communication problems or with previous documents of cognitive impairment such as dementia. The questionnaire we used was validated in first-ever stroke patients and is not specifically designed for the evaluation of knowledge in a high-risk population, that is, patients with recurrent stroke or recurrent TIA. Furthermore, the small sample size may have limited the ability to differentiate levels of knowledge between the different groups of patients.

## 5. Conclusions

The results showed that knowledge of stroke in patients with recurrent stroke or TIA is still unsatisfactory. The majority of patients with risk factors could not identify their own risk factors. More than one-tenth of patients had a problem identifying one stroke warning sign. The low level of knowledge found in this study clearly indicates that there is an urgent need to provide a national policy to improve educational system to provide adequate structured information to increase self-management in patients with recurrent stroke.

## Figures and Tables

**Table 1:** Characteristics and demographic data of patients (*n* = 140).

Characteristic	Value
Age (years)	65.6 ± 11.3 (range 26–87)
Male sex, *n* (%)	78 (55.7%)
Living situation, *n* (%)	
Living alone	7 (5.0%)
Living with others	133 (95.0%)
Family members	4.4 ± 1.5
Education, *n* (%)	
Less than or equal to secondary school	110 (78.6%)
Higher than secondary school	30 (21.4%)
Duration after recurrent stroke, median (IQR) (days)	3 (25.3)
Comorbidities, *n* (%)	
Hypertension	56 (40.0%)
Diabetes mellitus	56 (40.0%)
Dyslipidemia	34 (24.3%)
Ischemic heart disease	3 (2.1%)
Atrial fibrillation	1 (1.4%)
None	1 (1.4%)
Stroke type (2nd episode), *n* (%)	
TIA	14 (10.0%)
Ischemic stroke	119 (85.0%)
Hemorrhagic stroke	7 (5.0%)
Presenting symptoms for this episode	
Limb weakness	112 (80%)
Facial weakness	50 (35.7%)
Difficulty in communication	27 (19.3%)

Values are mean ± SD unless otherwise indicated *n*, number; SD, standard deviation; TIA, transient ischemic attack.

**Table 2:** Source of information, perception of knowledge, and area of lack of knowledge about stroke.

Outcome variable	*N* (%)
*Prior information gained about stroke*	
Yes	121 (86.4%)
No	19 (13.6%)
*Source of information*	
Health care professional: nurses	107 (76.4%)
Health care professional: doctors	97 (69.3%)
Printed documents	41 (29.0%)
Television	40 (28.6%)
Radio	31 (22.1%)
Newspaper	18 (12.9%)
*Perceived lack of knowledge*	
Very high lack of knowledge	6 (4.3%)
High lack of knowledge	20 (14.3%)
Moderate lack of knowledge	67 (47.9%)
Little lack of knowledge	30 (21.4%)
Very little lack of knowledge	17 (12.1%)
*Area of lack of knowledge*	
No lack of knowledge	20 (14.3%)
Do not know what is lacking	22 (15.7%)
Rehabilitation	28 (20.0%)
Diet	28 (20.0%)
Cause and clinical effects of stroke	27 (19.3%)
Exercise	24 (17.1%)
Medication	22 (15.7%)
Clinical course	19 (13.6%)
Treatment	19 (13.6%)
Psychological adjustment after stroke	18 (12.4%)
Stroke recurrence	17 (12.1%)
Complications after stroke	16 (11.4%)
How to be independent after stroke	15 (10.7%)
How to prevent recurrence	13 (9.3%)

**Table 3:** Knowledge of stroke risk factors and warning signs (open-ended questionnaire).

Study question	*N* (%)
*Definition of TIA*	
Unknown	62 (44.3%)
Inadequate blood supply to the brain/vessel occlusion	41 (29.3%)
Temporary inadequate blood supply to the brain	5 (3.6%)
Others (stress, weakness, etc.)	32 (22.8%)
*Definition of stroke*	
Unknown	45 (32.1%)
Ischemic brain/inadequate brain perfusion	48 (34.2%)
Muscle weakness	27 (19.3%)
Brain abnormalities	10 (7.1%)
Others (fatigue, dizziness, etc.)	8 (5.7%)
*Stroke caused by a disorder of*	
Unknown	34 (24.3%)
Brain	35 (25.0%)
Heart	16 (11.4%)
Muscle	10 (7.1%)
Underlying disease	17 (12.1%)
Behavioral problem	20 (14.3%)
*Risk factors of stroke*	
Unknown	35 (25.0%)
Hypertension	49 (35.0%)
Dyslipidemia	40 (28.6%)
Diabetes mellitus	32 (22.9%)
Stress	20 (14.3%)
Smoking	18 (12.9%)
Alcohol drinking	12 (8.6%)
Physical inactivity	9 (6.4%)
Aging	8 (5.7%)
Poor medical control	6 (4.3%)
Obesity	5 (3.6%)
Others but incorrect	9 (6.4%)
*Knowledge of stroke symptoms*	
Could not identify any	19 (13.6%)
Sudden unilateral weakness of face, arm, or leg	86 (61.4%)
Sudden trouble with speaking	36 (25.7%)
Sudden trouble with walking, loss of balance, or dizziness	30 (21.4%)
Sudden severe headache with no known causes	18 (12.9%)
Sudden unilateral numbness of face, arm, or leg	6 (4.3%)
Sudden communication problem	3 (2.1%)
Sudden trouble with seeing in one or both eyes	1 (0.07%)
Irrelevant symptoms	4 (2.9%)

**Table 4:** Knowledge of stroke risk factors and warning signs in patients (closed-ended questionnaire).

Question	Number of correct responses [*N* (%)]
*Definition of stroke and TIA (5 scores)*	
TIA is a temporary obstruction of blood supply to the brain	131 (93.6%)
TIA symptoms are usually of short duration, in minutes or a few hours	123 (87.9%)
TIA is a warning sign of stroke	120 (85.7%)
Stroke causes a neurological deficit that lasts longer than 24 hours	118 (84.3%)
Stroke is caused by the occlusion or rupture of brain vessels	132 (94.3%)
*Stroke risk factors (5 scores)*	
Hypertension	135 (96.4%)
Dyslipidemia	135 (96.4%)
Aging	132 (94.3%)
Diabetes	121 (86.4%)
Heart disease	119 (85.0%)
*Stroke warning signs (7 scores)*	
Sudden unilateral weakness of face, arm, or leg	138 (98.6%)
Sudden unilateral numbness of face, arm, or leg	137 (97.9%)
Sudden trouble with walking, loss of balance, or dizziness	129 (92.1%)
Sudden trouble with speaking	123 (87.9%)
Sudden severe headache with no known causes	111 (79.3%)
Sudden trouble with seeing in one or both eyes	96 (68.6%)
Sudden communication problem	94 (67.1%)

**Table 5:** Relation between stroke knowledge score (full score 17) and other variables.

Variables (*n*)	Stroke knowledge score (mean ± SD)	Mean difference (95% CI)	*p* value^a^
Gender			
Male (78)	14.6 ± 2.7	−0.76 (−1.6 to 1.6)	0.059
Female (62)	15.4 ± 2.8		
Age group			
≤60 years (38)	15.3 ± 2.3	0.4 (−0.6 to 1.4)	0.71
>60 years (102)	14.9 ± 2.9		
Educational level			
Lower than secondary school (110)	14.8 ± 2.9	−0.7 (−1.7 to 0.4)	0.22
Higher (30)	15.5 ± 2.0		
Living situation			
Living alone (7)	15.3 ± 3.3	0.3 (−1.8 to 2.5)	0.43
Living with others (133)	14.9 ± 2.7		
Barthel index			
≤60 (29)	13.9 ± 2.7	−1.1 (−2.2 to −0.04)	0.03
>60 (73)	15.1 ± 2.4		

*Note*. ^a^Mann–Whitney *U* test.
